# Soil carbon stocks in forest-tundra ecotones along a 500 km latitudinal gradient in northern Norway

**DOI:** 10.1038/s41598-022-17409-3

**Published:** 2022-08-03

**Authors:** Claire Céline Devos, Mikael Ohlson, Erik Næsset, Ole Martin Bollandsås

**Affiliations:** Department of Ecology and Natural Resource Management, Norwegian University of Life Sciences, P.O. Box 5003, 1432 Ås, NO Norway

**Keywords:** Carbon cycle, Boreal ecology, Carbon cycle, Forest ecology

## Abstract

As shrubs and trees are advancing into tundra ecosystems due to climate warming, litter input and microclimatic conditions affecting litter decomposition are likely to change. To assess how the upward shift of high-latitude treeline ecotones might affect soil organic carbon stocks (SOC), we sampled SOC stocks in the surface layers of 14 mountain birch forest-tundra ecotones along a 500 km latitudinal transect in northern Norway. Our objectives were to examine: (1) how SOC stocks differ between forest and tundra soils, and (2) the relative role of topography, vegetation and climate in explaining variability in SOC stock sizes. Overall, forest soils had higher SOC stocks (median: 2.01 kg m^−2^) than tundra soils (median: 1.33 kg m^−2^). However, SOC storage varied greatly within and between study sites. Two study sites had higher SOC stocks in the tundra than in the nearby forest, five sites had higher SOC stocks in the forest, and seven sites did not show differences in SOC stocks between forest and tundra soils. Thus, our results suggest that an upwards forest expansion does not necessarily lead to a change in SOC storage at all sites. Further, a partial least-squares regression (PLSR) model indicated that elevation, temperature, and slope may be promising indicators for SOC stock size at high-latitude treelines. Precipitation and vegetation were in comparison only of minor importance.

## Introduction

Boreal ecosystems store approximately one third of all terrestrial carbon (C)^1^, and thereby play an important role in the global carbon cycle. The majority of C in the boreal biome is found belowground, with particularly large amounts in peatlands and permafrost-affected mineral soils^2^. The large accumulation of soil organic carbon (SOC) in such ecosystems is mainly the result of slow decomposition rates due to low temperatures and anoxic conditions^3^. In addition, typical boreal vegetation, such as mosses and coniferous trees, tends to deposit litter that is quite resistant to decomposition because of its chemical composition^4^.

One of the most striking vegetation transitions in the boreal biome is the treeline ecotone. In Fennoscandia, this ecotone is a zone where boreal forests dominated by mountain birch (*Betula pubescens* ssp. *czerepanovii*), Norway spruce (*Picea abies*)*,* or Scots pine (*Pinus sylvestris*) give way to alpine or arctic tundra^5^. Here, considerable differences in soil C cycling may occur over short distances because of local-scale variability in terrain properties, climate and vegetation^6,7^. The predicted greater-than-average climate warming at high latitudes^8^ is likely to cause profound changes in SOC pools across the forest-tundra ecotone, and there is a concern that a substantial proportion of this SOC could be lost to the atmosphere in the future^9–11^.

Climate warming is likely to have both direct and indirect effects on SOC cycling in the treeline ecotone. For instance, experimental warming experiments have shown that even small changes in temperature could enhance decomposer activity and increase CO_2_ efflux from both forest and tundra soils^12,13^. In addition to direct warming effects, climate change may also impact treeline SOC dynamics indirectly through shifts in the distribution of vegetation communities. Numerous studies have reported an upward and/or northward advance of boreal forests at the expense of tundra ecosystems since 1900 AD^14–16^. Global warming is widely recognized as the main driver, although it should be noted that local-scale treeline dynamics are mediated by several additional biotic and abiotic factors^17–19^. In the Scandes mountains, treelines have for instance greatly been affected by anthropogenic activities such as the use of upland pastures for livestock grazing and fire wood collection^20,21^. A shift from tundra to forest is likely to impact SOC storage, but the magnitude of such a response remains unclear.

The encroachment of forest species into tundra communities will not only alter C input into the ecosystem by increasing aboveground plant biomass and litter, but may also impact C output by creating a more favorable microclimate for decomposition (e.g. increased snow accumulation and soil temperatures)^6^. It has also been argued that increased decomposition rates could enhance the mineralization of nitrogen and other soil nutrients that commonly limit plant productivity in boreal ecosystems. Enhanced plant productivity could in turn increase C sequestration, and potentially compensate for C losses via respiration^9,22,23^.

To be able to predict how treeline SOC dynamics may respond to climate change, it is crucial to quantify the size of current SOC stocks and identify the key drivers behind their accumulation. Only very few studies have examined SOC dynamics above the treeline, and these suggest that organic surface soil horizons in the tundra contain larger SOC pools compared to those of the adjacent forest^12,24–27^. Conflicting results have been reported for deeper situated mineral soil horizons^24–27^. The high spatial variability that characterizes treeline ecotones also suggests that SOC stocks may vary substantially across very short distances. At a local scale, for example, topographic features may affect the spatial distribution of SOC stocks by controlling the flow of water and organic matter depositions. Lower slopes and concave curvatures tend to have higher SOC pools because they receive surface runoff and sediments from the surroundings. Convex curvatures and steep slopes, on the other hand, tend to lose organic matter as the topsoil is constantly eroded^28^. Slope aspect, defined as the direction a slope faces, might affect the received solar radiation intensity and create a microclimate that differs considerably from regional climatic conditions^29^. Vegetation community structure and composition is likely to be another key driver behind the accumulation of SOC through differences in litter production and its decomposability^4,30,31^, and SOC stocks are known to vary under the different treeline-forming species found in the Scandes mountains^32^.

Treeline ecotones in the northern Scandes mountains are characterized by sharp borders between mountain birch forests and tundra areas^5^, which in turn encompass marked changes in vegetation, microclimate, and topography across fine spatial scales. Here we have quantified SOC stocks in 14 forest-tundra ecotones along a 500 km latitudinal gradient in northern Norway, and we hereby present results from the currently largest field study on SOC storage in forest-tundra ecotones. The wide latitudinal extent enables examinations of possible regional differences in SOC as a result of precipitation- and temperature variability, and local variations in vegetation and microtopography. Our objectives were to examine: (1) how SOC stocks differ between forest and tundra soils, and (2) the relative role of topography, vegetation, and climate in explaining variability in SOC stock sizes. We focused on SOC in the surface soil (O and A horizons), as these horizons are rich in organic matter and have a high potential for rapid changes in C sequestration under climate change. In line with previous studies that quantified SOC stocks in the organic soil horizons at high-latitude treelines, we expected SOC stocks to be higher in tundra soils above the treeline than in adjacent forest soils.

## Materials and methods

### Study sites

**Figure 1:**
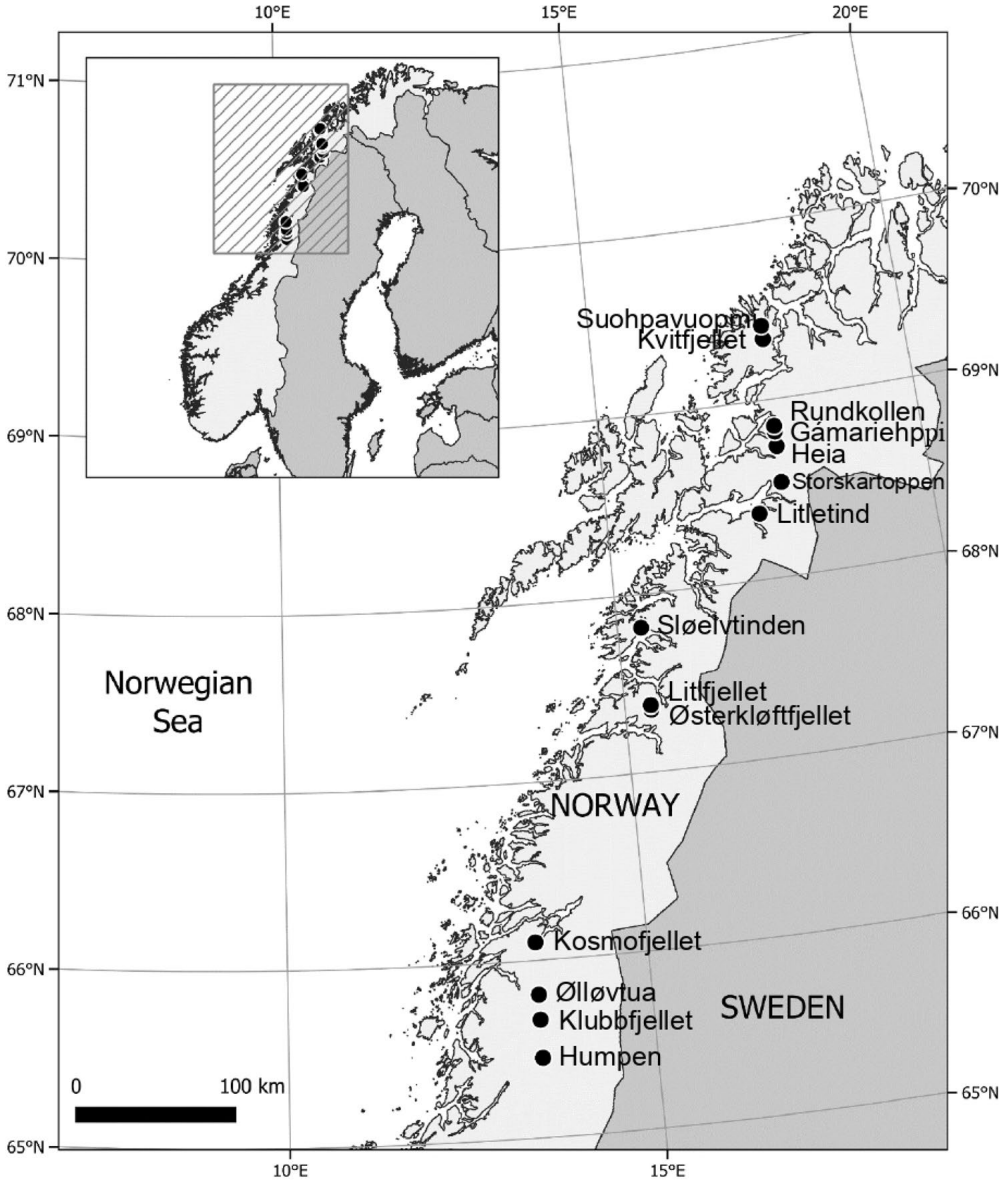
Map showing the 14 study sites located along a 500 km latitudinal gradient in northern Norway.

This study was carried out along a 500 km long latitudinal transect across Norway that was part of a 1100 km long latitudinal transect originally established by Thieme et al*.*^33^. The study sites (~ 50 m × 200 m) along the transect are forest-tundra ecotones selected for their proximity to sample plots used in Norway’s National Forest Inventory. For this study, we revisited the 14 northernmost sites (Fig. 1, Table 1). At all 14 sites, mountain birch (*Betula pubescens* spp. *czerepanovii*) is the dominating tree species*.* The field- and ground layer vegetation vary considerably within and between sites due to highly variable topography and climatic conditions, although it typically consists of a mix of alpine and boreal forest species, among which *Betula nana, Salix* spp*., Vaccinium* spp., *Empetrum nigrum, Rubus chamaemorus, Calluna vulgaris*, *Carex* spp., and *Eriophorum* spp. are common in the field layer, and *Hylocomium splendens*, *Pleurozium schreberi*, *Sphagnum* spp., *Cladonia* spp., and *Flavocetraria* spp*.* are common in the ground layer. Detailed information about the vegetation at our study sites is given by Mienna et al.^34^.

**Table 1:** Location and characteristics of the 14 study sites.

Site	Latitude	Longitude	Elevation (m a.s.l.)	MT (°C)	MST (°C)	MP (mm)	MSP (mm)	MR (Wm^2^ s^−1^)	MSR (Wm^2^ s^−1^)	GS (days)	Slope (%)	Aspect
Humpen, Grane	65° 27′ 01″ N	13° 26′ 38″ E	449–478	1.71	9.58	5.37	4.20	6.24	8.48	130	11.3	158
Klubbfjellet, Grane	65° 39′ 59″ N	13° 26′ 45″ E	562–606	1.27	8.79	5.16	4.03	6.26	8.55	121	10.0	219
Ølløvtua, Mosjøen	65° 48′ 21″ N	13° 26′ 50″ E	614–646	1.19	8.40	7.42	5.85	6.28	8.58	115	12.3	145
Kosmofjellet, Fauske	67° 21′ 09″ N	15° 22′ 59″ E	467–482	2.29	9.25	5.67	4.55	6.25	8.52	130	19.1	176
Østerkløftfjellet, Fauske	67° 22′ 34″ N	15° 22′ 55″ E	524–564	2.05	8.71	5.00	4.65	6.24	8.53	123	20.3	234
Litlfjellet, Mosjøen	66° 06′ 09″ N	13° 26′ 56″ E	465–520	2.17	8.88	6.03	5.84	6.24	8.53	126	16.0	294
Sløelvtinden, Steigen	67° 48′ 53″ N	15° 21′ 11″ E	441–441	2.95	9.31	4.20	3.69	6.20	8.47	135	2.5	168
Litletind, Narvik	68° 22′ 15″ N	17° 19′ 44″ E	589–635	1.65	8.13	4.04	3.99	6.37	8.77	114	19.7	142
Storskartoppen, Narvik	68° 31′ 57″ N	17° 43′ 33″ E	411–441	1.73	9.11	4.35	4.18	6.35	8.71	124	15.6	199
Heia, Lavangen	68° 44′ 03″ N	17° 44′ 04″ E	445–518	1.10	8.56	4.38	3.86	6.30	8.70	119	11.0	104
Gámariehppi, Lavangen	68° 49′ 02″ N	17° 44′ 14″ E	522–584	0.80	8.03	3.83	3.38	6.29	8.69	111	15.2	121
Rundkollen, Salangen	68° 50′ 53″ N	17° 44′ 20″ E	477–563	0.91	8.15	5.02	4.42	6.29	8.68	113	22.4	187
Kvitfjellet, Senja	69° 20′ 26″ N	17° 45′ 30″ E	309–348	1.88	8.70	4.09	3.34	6.28	8.63	123	18.3	55
Suohpavuopmi, Senja	69° 25′ 03″ N	17° 45′ 41″ E	324–346	2.04	8.59	4.93	3.97	6.28	8.60	123	8.2	241

*MT* mean temperature, *MST* mean summer temperature, *MP* mean precipitation, *MSP* mean summer precipitation, *MR* mean solar radiation, *MSR* mean summer solar radiation, *GS* mean length of the thermal growing season. Reference period climate data: 1970–2020.

### Soil sampling and analysis

At each of the 14 sites, we set up two soil sample transects in the forest and one soil sample transect in the tundra. All sample transects had a north–south orientation and consisted of 10 sample points. In the forest, we established each of the two sample transects centered around a mountain birch tree. The two mountain birch trees were located at least 10 m apart. Sample points were selected 0.5, 1.2, 1.6, 2.4 and 4 m northward and southward from the tree trunks. In the tundra, we set up one similar sample transect without a center point tree, but rather centered around a point located on the western border of each study site, 10 m southward from the top. The distance between the forest and tundra sample transects was ~ 200 m.

Soil core samples including the entire organic surface soil (i.e., O and A horizons) were collected at each sample point using a cylindrical soil sampler (Ø = 6.35 cm) during August and September 2020. As there was typically no clear border between the O- and A horizons, we did not attempt to separate these soil layers. Therefore, the two horizons were bulked and only the thickness of the entire OA complex was measured. The samples were frozen at − 20 °C until further analysis. To get precise coordinates of the soil sample points, we used a Topcon HiPer SR receiver in real-time kinetic mode, receiving differential corrections of both the Global Navigation Satellite System and the Global Positioning System.

In the laboratory, the samples were defrosted and fresh plant litter such as leaves and twigs that had not undergone observable decomposition, was removed. The soil samples were dried to constant mass at 40 °C in a drying cabinet. Bulk densities were determined based on the dry matter mass and sample volume. The dried samples were milled and homogenized. Total SOC concentrations were determined by dry combustion using a vario MICRO cube element analyser (Elementar, Hanau, Germany). Volume-based SOC stocks were calculated as:

1 $$\begin{aligned} {\text{SOC}}\;{\text{stock}}\left( {{\text{kg}}\;{\text{m}}^{ - 2} } \right) & = {\text{bulk}}\;{\text{density}}\left( {{\text{g}}\;{\text{cm}}^{ - 3} } \right) \times {\text{soil}}\;{\text{depth}}\left( {{\text{cm}}} \right) \, \\ & \quad \times {\text{SOC}}\;{\text{concentration}}\left( \% \right) \times 0.1 \\ \end{aligned}$$

### Environmental variables

Data on topographic, vegetation and climatic variables that potentially control the storage of SOC were collected in the field or extracted from existing databases. Topographic attributes (elevation, slope, and aspect) were derived from digital elevation models with a resolution of 1 m provided by the Norwegian Mapping Authority using QGIS version 3.16.8. The aspect values were cosine and sine transformed to obtain continuous variables that represent north–south and east–west orientation. To avoid 0 values, we added 2 to both variables, thus obtaining values ranging between 1 (south) and 3 (north) for cos(aspect) and values between 1 (west) and 3 (east) for sin(aspect). In the field, each sample point was assigned a vegetation class (vascular plants, *Sphagnum* mosses, other mosses, or lichens) based on the dominant species within a surrounding circular plot (Ø = 60 cm) around the sample point.

Climatic variables were calculated on the study site-level based on daily weather estimates interpolated from Norway’s official weather stations by the Norwegian Meteorological Institute^35^. We calculated mean daily temperature, precipitation, and solar radiation for the period between 1970 and 2020. We also calculated mean summer temperature, precipitation, and solar radiation as the mean of the daily observations within the period from June 1st to September 30th. In addition, we calculated the length of the thermal growing season. Since 5 °C is a commonly accepted thermal threshold for vegetation growth in the Nordic region^36^, we defined the thermal growing season as the period with a mean daily temperature equal to or above 5 °C.

### Statistical analyses

Statistical analyses were carried out using R (R Core Team, 2021). First, we analyzed how SOC stocks differ between forest and tundra soils. All continuous variables were tested for normality using Shapiro–Wilk tests. Due to the non-normal distributed nature of our dataset, non-parametric Mann–Whitney *U* tests were used to assess differences in soil properties (soil depth, SOC concentration, and SOC stock) between forest and tundra soils. Differences in SOC stocks between vegetation classes were analyzed with a Kruskal–Wallis test followed by a Dunn test with Bonferroni correction of *p-*values.

To evaluate the importance of environmental variables as determinants of SOC stocks, we started by calculating Spearman’s rank correlation coefficients among all continuous predictor variables and SOC stocks. Due to strong intercorrelations between several variables, they could not be considered as independent determining factors. To cope with this multicollinearity problem, we used partial-least squares regression (PLSR). PLSR is a multivariate analysis method used to explain variation in one or several response variables by constructing linear combinations of predictor variables. Hence, PLSR is a particularly suitable method to analyze datasets with numerous, highly correlated predictor variables. A detailed mathematical description of PLSR can be found in Abdi^37^ and Wold et al.^38^.

We built a PLSR model using the R package *pls*^39^. Before setting up the model, all continuous variables were standardized (mean = 0, SD = 1) to give them the same prior importance. The dependent variable (SOC stock) was log-transformed to get a normal distribution. To avoid overfitting, the number of components included in the final model was determined systematically using the one-sigma heuristic method with leave-one-out (LOO) cross validation. LOO cross validation consists of excluding each sample observation once, constructing a model without this observation, and predicting the value of the dependent variable. The residuals (i.e., the difference between observed and predicted values) are used to calculate the cross-validated root mean square error of cross validation (RMSEP_CV_). The one-sigma heuristic method selects the smallest number of components still capable of producing a RMSEP_CV_ that falls within one standard error of the absolute minimum RMSEP_CV_^40^. Two important statistics that describe the performance of the final model are the explained variation in the response (R^2^) and the predicted variation in the response (Q^2^, i.e., goodness of prediction, or cross-validated R^2^). A large difference between R^2^ and Q^2^ indicates that the model suffers from overfitting. In PLSR modelling, the relative influence of each predictor variable can be given by the variable importance in projection (VIP), which is the sum of the variable’s influence over all model dimensions divided by the total variation explained by the model. Variables with VIP scores ≥ 1 are generally considered to be the most relevant for explaining variability in the dependent variable. The regression coefficients (RC) reveal the direction of the influence of each variable^41,42^.

### Ethical statement

Experimental research and field studies on plants (either cultivated or wild), including the collection of plant material, complies with relevant institutional, national, and international guidelines and legislation.

## Results

### SOC stocks in high-latitude treeline surface soils

**Figure 2:**
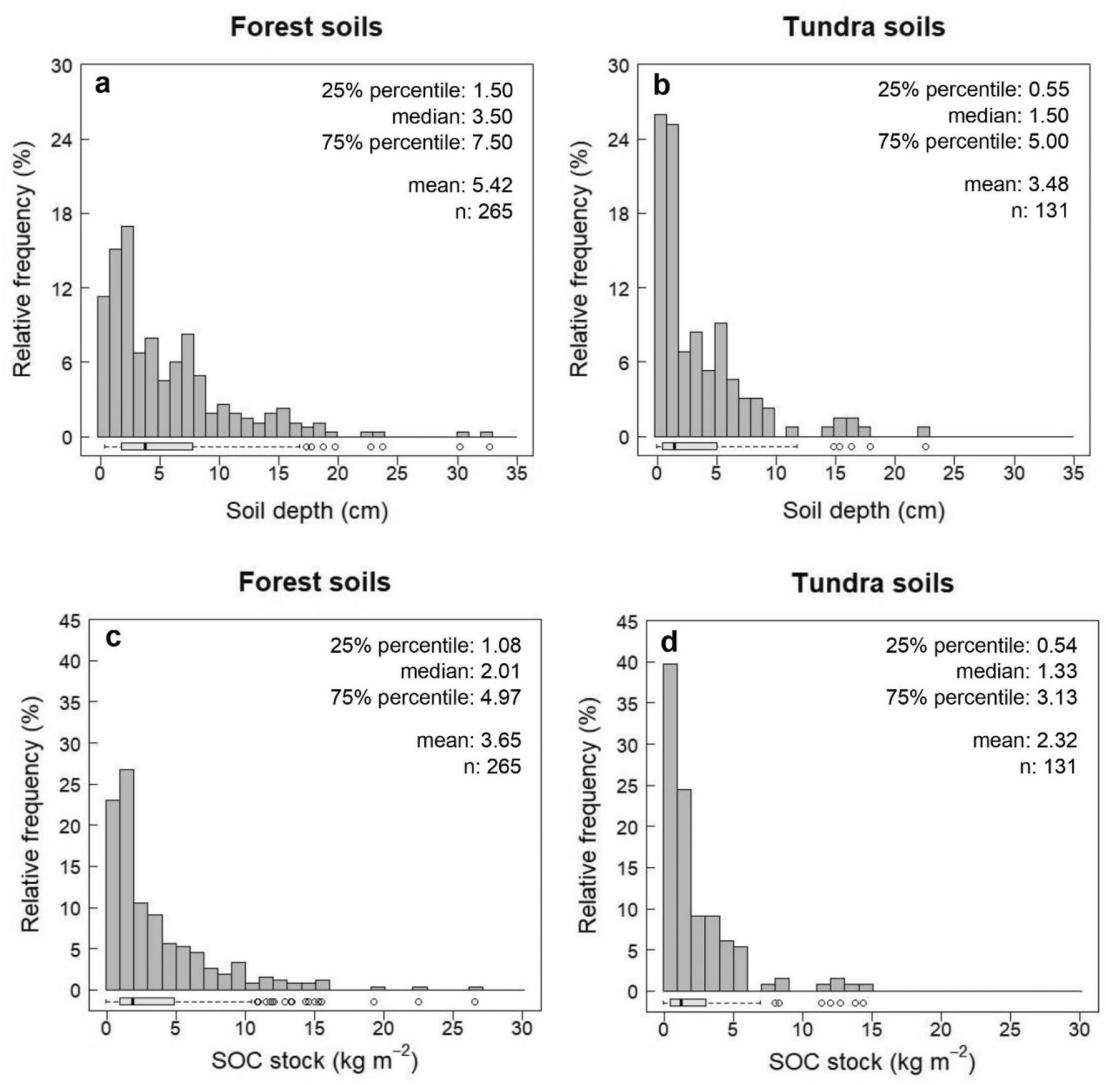
Soil depths and soil organic carbon (SOC) stocks of surface soils across 14 treeline ecotones in northern Norway. Soil depths of forests at treeline (**a**), soil depths of tundra above the treeline (**b**), SOC stocks of forests at treeline (**c**), SOC stocks of tundra above the treeline (**d**).

**Figure 3:**
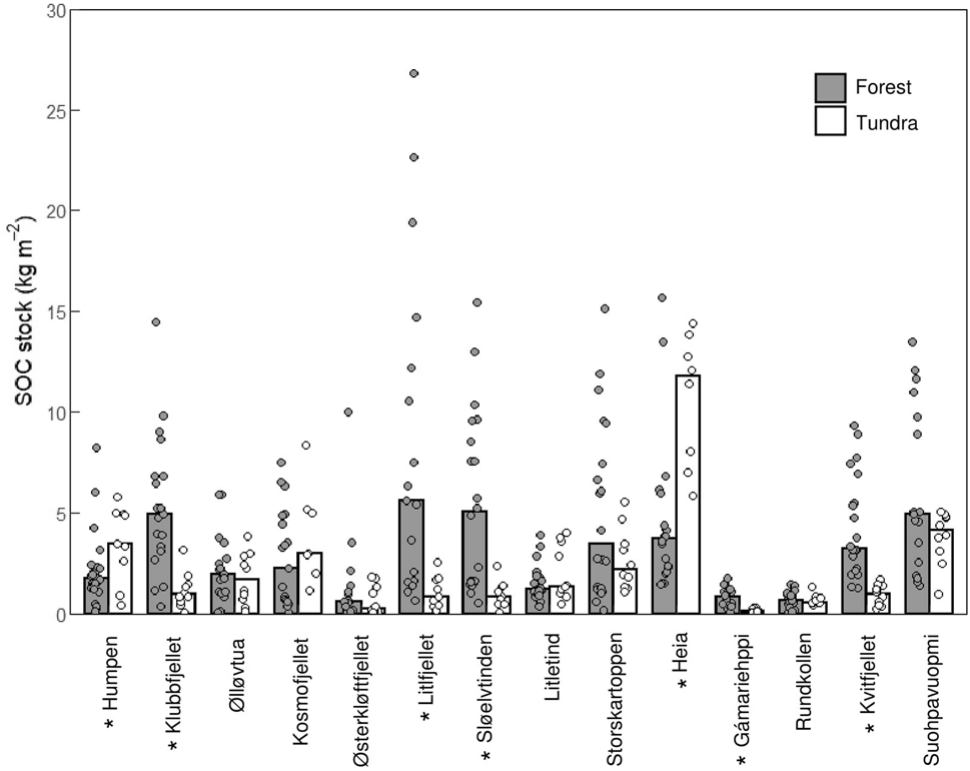
Soil organic carbon (SOC) stocks in surface soils in treeline forests and in tundra above the treeline across 14 treeline ecotones in northern Norway. Bars show median values, datapoints show individual observations. Study sites are arranged from southernmost site (i.e., Humpen) to northernmost site (i.e., Suohpavuopmi). Study sites with statistically significant (Mann–Whitney *U* tests, *p* < 0.05) differences between SOC stocks in forest and tundra soils are indicated with an asterisk.

Soil characteristics varied substantially across the treeline ecotone. Overall, the sampled soil horizons were significantly thicker (*p* < 0.001) under mountain birch canopies in the forest (median: 3.50 cm) than in the tundra (median: 1.50 cm) (Fig. 2a,b). Forest soils also had greater (*p* < 0.001) SOC concentrations (median: 44.0%) than tundra soils (median: 41.3%). Forest SOC stocks ranged from 0.04 up to 26.8 kg m^−2^ with a mean of 3.65 kg m^−2^. The median value was only 2.01 kg m^−2^, indicating a highly skewed distribution of forest SOC stocks (Fig. 2c). Tundra SOC stocks were significantly smaller than forest SOC stocks (*p* < 0.001) and covered a narrower range (0.05–14.4 kg m^−2^). Tundra SOC stocks also showed a right-skewed distribution, with most soils being characterized by small SOC stocks (mean: 2.32 kg m^−2^, median: 1.33 m^−2^) (Fig. 2d). Moreover, soil characteristics varied greatly within and between study sites. An overview of soil depths and SOC concentrations of forest and tundra soils at each of the study sites can be found as Supplementary Table S1. Out of the 14 study sites, five had greater SOC stocks in the forest than in the nearby tundra (*p* < 0.01). Two sites had greater SOC stocks in the tundra than in the forest (*p* < 0.05) (Fig. 3). Vegetation communities dominated by *Sphagnum* spp. had particularly large soil thicknesses (median: 8.05 cm) and stored large amounts of SOC (median: 3.89 kg m^−2^). In comparison, the other vegetation classes exhibited shallower soil layers (median: 2.25–3.50 cm) with significantly smaller SOC stocks under vascular plants (median: 1.86 kg m^−2^, *p* < 0.05), other mosses (median: 1.40 kg m^−2^, *p* < 0.01), and lichens (median: 1.34 kg m^−2^, *p* < 0.01).

### Topographic, climatic and vegetation controls of treeline SOC stocks

To identify which variables control the accumulation of SOC stocks in treeline soils, we computed a correlation matrix including different topographic and climatic variables (Table 2). SOC stocks showed highly significant correlations (*p* < 0.01) with elevation, mean temperature, mean summer temperature, slope, length of the thermal growing season, and east–west slope aspect. However, many of these variables were inter-correlated and could therefore not be treated as independent determining factors of SOC variability. Thus, we built a PLSR model to extract and rank the most important factors controlling SOC stocks.

**Table 2:** Correlation matrix of SOC stocks with climatic and topographic variables.

	SOC	Latitude	Elevation	MT	MST	MP	MSP	MR	MSR	Slope	GS	Cos(aspect)
Latitude	0.014											
Elevation	− 0.330**	− 0.465**										
MT	0.201**	− 0.160**	− 0.406**									
MST	0.245**	− 0.577**	− 0.383**	0.692**								
MP	0.075	− 0.621**	0.153**	0.145**	0.371**							
MSP	− 0.115*	− 0.584**	0.367**	0.120*	0.193**	0.842**						
MR	− 0.006	0.583**	0.028	− 0.593**	− 0.692**	− 0.521**	− 0.320**					
MSR	− 0.038	0.592**	0.061	− 0.607**	− 0.722**	− 0.553**	− 0.313**	0.986**				
Slope	− 0.178**	0.070	0.120*	0.022	− 0.140	− 0.036	0.189**	0.137	0.144**			
GS	0.284**	− 0.422**	− 0.504**	0.833**	0.946**	0.298**	0.138**	− 0.694**	− 0.724**	− 0.166**		
Cos(aspect)	0.132*	0.419**	0.166**	− 0.409**	− 0.626**	− 0.242**	− 0.177**	0.539**	0.540**	0.226**	− 0.616**	
Sin(aspect)	− 0.203**	0.189**	− 0.092	− 0.231**	− 0.116*	− 0.345**	− 0.375**	0.277**	0.226**	0.099	− 0.172**	0.037

*SOC* soil organic carbon stock (kg m^−2^), *MT* mean temperature (°C), *MST* mean summer temperature (°C), *MP* mean precipitation (mm), *MSP* mean summer precipitation (mm), *MR* mean solar radiation (Wm^2^ s^−1^), *MSR* mean summer solar radiation (Wm^2^ s^−1^), *GS* mean length of the thermal growing season (days).

*Level of significance: p < 0.05.

**Level of significance: p < 0.01.

**Figure 4:**
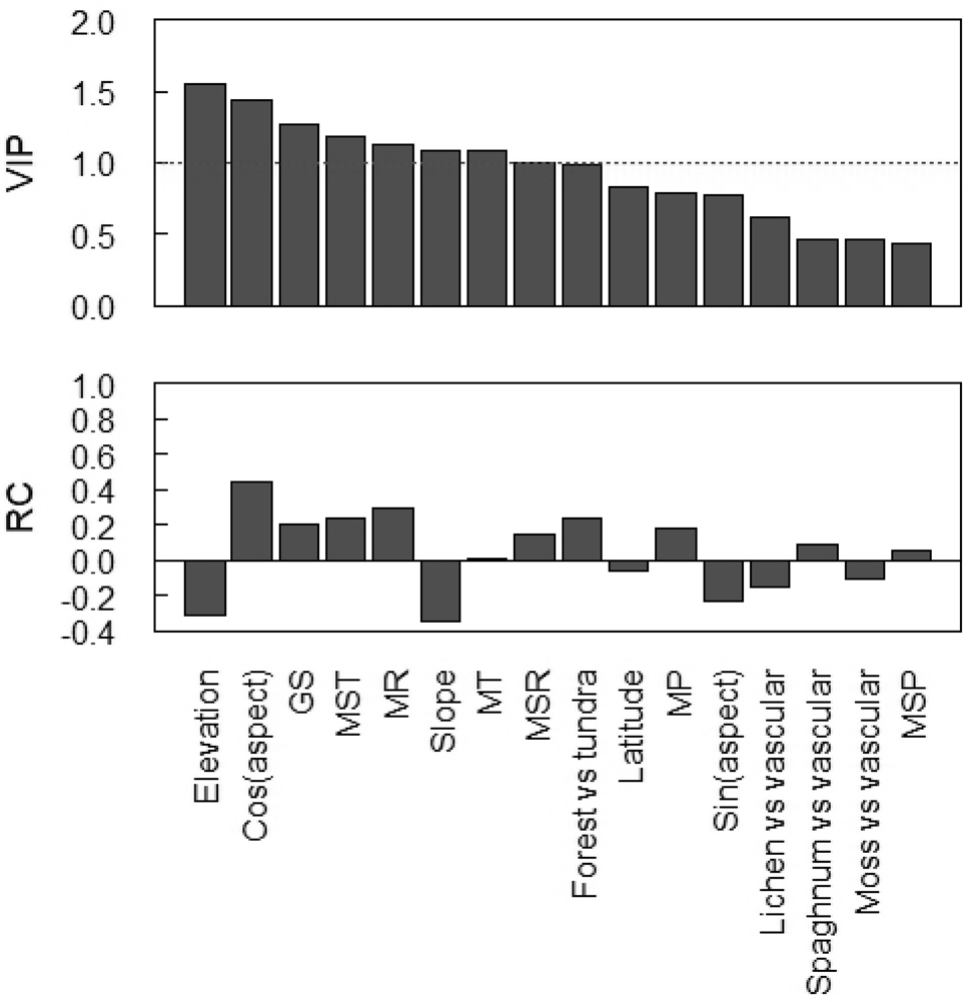
Variable importance in projection (VIP) values and regression coefficients (RC) of the explanatory variables included in the PLSR model. The dotted line indicates a VIP threshold above which predictors are considered important. Cos(aspect): south-north slope aspect, GS: mean length of the thermal growing season (days), MST: mean summer temperature (°C), MR: mean solar radiation (Wm^2^ s^−1^), MT: mean temperature (°C), MSR: mean summer solar radiation (Wm^2^ s^−1^), MP: mean precipitation (mm), Sin(aspect): west–east slope aspect, MSP: mean summer precipitation (mm).

Our modelling approach resulted in a final PLSR model with three components (Table 3). The model had a moderate explanatory power accounting for 38.6% of the variability in SOC stocks. The corresponding cross-validated R^2^ cum (Q^2^ cum) of 34.4% indicates that the model does not suffer from overfitting. Figure 4 illustrates the VIP values and regression coefficients. Elevation was the most important determinant of SOC stock variability, followed by north–south slope aspect, mean length of the thermal growing season, mean summer temperature, mean solar radiation, slope, mean temperature, and mean summer solar radiation. All other variables had a VIP value < 1 and were thereby of minor importance in explaining variability in SOC stocks. As indicated by the regression coefficients, SOC stocks decreased with increasing elevation and slope, while they increased with increasing temperature and solar radiation. SOC stocks were higher on north-oriented slopes than on south-oriented slopes.

**Table 3:** Summary of the partial least square regression (PLSR) model for soil organic carbon (SOC) stocks (kg m^−2^) in treeline soils.

Component	R^2^ (%)	R^2^ cum (%)	Q^2^ (%)	Q^2^ cum (%)	RMSEP_CV_
1	21.1	21.1	17.6	17.6	1.13
2	12.5	33.6	11.5	29.1	1.05
3	5.0	38.6	5.2	34.3	1.01

*R*^*2*^ explained variability, *Q*^*2*^ cross-validated R^2^, *cum* cumulative, *RMSEP*_*CV*_ cross-validated root mean square error of prediction.

## Discussion

### SOC stock sizes across the treeline ecotone

As boreal forests are currently advancing into tundra regions at many locations^18^, it is important to understand how SOC dynamics differ between forest and tundra soils. In this study, we sampled 14 treeline ecotones along a 500 km latitudinal gradient in northern Norway. Contrary to our expectations, our results indicate that SOC stocks in the surface soil (O and A horizons) are generally greater in mountain birch forests than in the tundra above the treeline. However, this general pattern does not hold true for all the study sites. Two study sites had higher SOC stocks in the tundra than in the nearby forest, while seven sites did not show differences in SOC storage between forest and tundra soils.

At present, there is no consensus on how the vegetation transition across the treeline ecotone affects SOC storage. Overall, there are three key predictions^11,43^ that may explain why we did not observe the same pattern at all study sites. First, carbon cycle models predict that the decrease in aboveground biomass and litter fall along the transition from forest into generally low-productive tundra results in smaller tundra SOC pools^44^. Second, previous field studies in the Scandes mountains suggest that SOC stocks in the organic horizon are smaller beneath mountain birch forest soils than beneath tundra soils due to higher soil respiration rates^12,25–27^. One commonly proposed explanation is that shrubs and trees trap more snow, which insulates the soil and thereby increases microbial activity^45,46^. Parker et al*.*^31^, however, found the effect of snow cover to be small, and suggested that variation in decomposition rates can rather be explained by litter quality and activity of the soil microbial community during summer. Third, forest and tundra soils may have similar SOC stocks if higher litter inputs in the forest are counterbalanced by accellerated decomposition rates. This hypothesis has been suggested for a forest-tundra ecotone in the Ural mountains, where total SOC stocks did not vary across the treeline despite differences in litter decomposibility^24^. Similar results have been found in a long-term warming experiment, where tussock tundra changing into shrub tundra increased the decomposer activity in the mineral soil horizon without altering SOC pools^47^.

Our large-scale dataset demonstrates that SOC stocks do not always decrease from the forest to the tundra as has previously been reported for surface soils in mountain birch forest-tundra ecotones ^6,25–27^. The contrasting results between different study sites strongly suggest that changes in SOC stock sizes across the treeline depend on the extent to which local environmental conditions alter the balance between litter input and soil respiration. It should be noted that we reported SOC stocks for the entire OA complex, while earlier work gave estimates for individual soil layers ^12,25–27^. Thus, direct comparisons of SOC stock sizes with previous studies should be done with caution because of methodological differences. To get a full picture of SOC dynamics at the forest-tundra ecotone, soil layers should ideally be sampled down to the bedrock. However, as the organic soil horizon contains the most labile and temperature-sensitive SOC^7^, our study is a valuable contribution to the scarce data on SOC stock sizes across high-latitude treeline ecotones.

### Within- and between site variability in SOC stock sizes

Since the quantification of SOC stock sizes through field studies at remote mountainous areas is cost- and time-intensive, the identification of variables controlling SOC storage and their incorporation into prediction models could be a promising alternative. Using PLSR, we ranked the relative importance of a few variables in explaining the high within- and between site variability in SOC stocks. Note that we included variables measured at two spatial scales. Climatic variables were measured at the study site level, while information on topography and vegetation was derived for each of the individual soil sample points.

Among the considered variables, elevation was the most important determinant of SOC stock size. In previous studies, elevation has often be found to be a good predictor of SOC as it integrates the effects of temperature and precipitation, and reflects erosional and depositional processes^48^. Our model, however, did not assign substantial explanatory power to precipitation (i.e., mean precipitation and mean summer precipitation). Temperature-related variables (i.e., south-north slope aspect, length of the thermal growing season, mean summer temperature, mean solar radiation, mean temperature, mean summer solar radiation), on the other hand, were considered to be of major importance in explaining SOC stock size variability. Overall, high elevations were negatively correlated with temperature and SOC stocks. Although low temperatures reduce microbial activity, and consequently limit decomposition, the increase of freezing episodes and duration of snow cover also limit plant productivity^49^. Thus, the low SOC stocks found at high elevations suggest that plant productivity may be the main driver of SOC accumulation in treeline soils. Moreover, the decrease in SOC with elevation may be associated with soil erosion along the hillslope of each study site. Within each site, sample points at high elevations were typically located at shoulderslope and backslope positions, while sample points at lower elevations were situated at footslope positions. Lower slope positions receive water runoff and sediments from upper slope positions, and may thus accumulate more SOC^28,29^. Easily derived from digital elevation models and meteorological datasets, elevation, temperature and slope seem to be promising indicators for SOC stock size at treeline ecotones.

Treeline ecotones form a mosaic of vegetation communities associated with nutrient availability, snow depth, wind-, and drainage conditions^50^. In this study, we sampled a wide variety of vegetation types, ranging from the edge of poorly-drained grass bogs to extremely dry and wind-exposed lichen patches. We therefore considered vegetation as a potential driver of SOC stock size variability. Although our PLSR model suggested that vegetation was only of minor importance in predicting SOC stocks compared with climate and topography, a Kruskal–Wallis test did reveal differences in SOC storage among vegetation types. Sample points dominated by *Sphagnum* spp. had particularly high SOC stocks, which may be explained by their high water retention capacity, low litter quality, and anoxic soil environments^51^.

### Implications of a shifting treeline for SOC stocks

Patterns in SOC stocks along the current vegetation transition from the forest to the tundra may hold clues regarding the consequences of future upwards treeline shifts^25^. Assuming that our comparison between adjacent forest and tundra soils represents a plausible ‘space-for-time’ approach, our data suggest that changes in SOC storage in response to treeline shifts will be highly spatially variable due to local site characteristics. The future storage of SOC in treeline soils, however, is impacted by a large number of interacting biotic and abiotic factors, several of which we did not take into account. For instance, SOC stocks will be impacted by direct effects of warming on decomposition^12^. Also, the response of SOC dynamics to treeline shifts is likely to change as the forest matures^26^. However, since we sampled 14 study sites, of which half had similar SOC stock sizes in forest and tundra soils, our study provides strong evidence that a shifting treeline does not necessarily lead to a change in SOC storage at all sites.

## Conclusion and outlook

Climate change is driving upward shifts of boreal forests into currently treeless tundra, and there is considerable concern that this vegetation shift may provide a positive feedback (i.e. further strengthen warming) on the global climate by reducing carbon storage. In our study covering 14 forest-tundra ecotones, only two sites had higher SOC stocks in the tundra than in the forest. Thus, we predict that the upwards advance of mountain birch forest into tundra communities does not necessarily lead to a decrease in SOC storage at all sites. Further, our results suggest that elevation, temperature, and slope may be promising indicators for SOC stock size. We strongly recommend future studies to continue identifying remotely measurable biotic and abiotic indicators of SOC stock size in forest-tundra ecotones, and we are convinced that remote sensing techniques represent highly promising alternatives to the regular monitoring of SOC stocks through time consuming and resource demanding field- and laboratory work.

## Supplementary Information

Supplementary Table S1.

## Data Availability

The dataset generated during the current study is available from the corresponding author on reasonable request.
