# Supplementary material for: Soil carbon stocks in forest-tundra ecotones along a 500 km latitudinal gradient in northern Norway
Source: Sci Rep. 2022 Aug 3;12:13358. doi: 10.1038/s41598-022-17409-3 (PMC9349290; doi:10.1038/s41598-022-17409-3)
Supplement: Supplementary file 1 — Supplementary Table S1. [file 41598_2022_17409_MOESM1_ESM.docx]

**Soil carbon stocks in forest-tundra ecotones along a 500 km latitudinal gradient in northern Norway**

Claire Céline Devos, Mikael Ohlson, Erik Næsset, Ole Martin Bollandsås

**Supplementary Table S1.** Soil depth, bulk density, SOC concentration and SOC stocks of surface soils in treeline forests and in the tundra above the treeline across 14 treeline ecotones in northern Norway. Values shown are median values, with range in parentheses. SOC: soil organic carbon.

|  | Forest soils | | | | Tundra soils | | | |
| --- | --- | --- | --- | --- | --- | --- | --- | --- |
| Site | Soil depth (cm) | Bulk density (g cm^-3^) | SOC (%) | SOC (kg m^-2^) | Soil depth (cm) | Bulk density (g cm^-3^) | SOC (%) | SOC (kg m^-2^) |
| Humpen,  Grane | 3.5  (0.3 - 11.5) | 0.11  (0.05 - 0.24) | 47.1  (36.9 - 49.0) | 1.8  (0.1 - 8.3) | 4.0  (0.2 - 8.5) | 0.43  (0.22 - 1.55) | 25.4  (17.9 - 42.3) | 3.5  (0.5 - 5.8) |
| Klubbfjellet, Grane | 8.0  (0.5 - 22.5) | 0.12  (0.07 - 0.26) | 46.4  (42.1 - 49.6) | 5.0  (0.4 -14.5) | 1.0  (0.1 - 6.0) | 0.26  (0.17 - 0.48) | 37.2  (20.1 - 47.0) | 1.0  (0.1 - 3.2) |
| Ølløvtua, Mosjøen | 2.0  (0.2 - 9.5) | 0.16  (0.05 - 0.32) | 43.7  (32.0 - 48.0) | 2.0  (0.0 - 5.9) | 1.8  (0.1 - 5.5) | 0.33  (0.15 - 1.54) | 31.3  (9.5 - 40.8) | 1.7  (0.2 - 3.9) |
| Kosmofjellet, Fauske | 4.5  (0.1 - 8.5) | 0.14  (0.04 - 0.46) | 44.3  (19.2 - 49.4) | 2.3  (0.1 - 7.5) | 5.5  (1.5 - 16.0) | 0.15  (0.08 - 0.48) | 38.2  (29.7 - 46.5) | 3.0  (1.2 - 8.4) |
| Østerkløftfjellet, Fauske | 1.0  (0.2 - 8.5) | 0.11  (0.06 - 0.35) | 36.8  (21.6 - 48.0) | 0.6  (0.1 - 10.0) | 0.8  (0.2 - 5.0) | 0.09  (0.06 - 0.13) | 43.8  (37.0 - 47.3) | 0.3  (0.1 - 1.8) |
| Litlfjellet, Mosjøen | 7.5  (1.0 - 32.5) | 0.19  (0.08 - 0.35) | 45.03  (35.3 - 49.8) | 5.6  (0.7 - 26.8) | 1.0  (0.3 - 5.6) | 0.26  (0.08 - 0.42) | 35.0  (24.2 - 45.7) | 0.9  (0.2 - 2.5) |
| Sløelvtinden, Steigen | 6.5  (0.4 - 17.1) | 0.19  (0.07 - 0.32) | 44.6  (27.2 - 50.2) | 5.1  (0.6 - 15.4) | 1.5  (0.5 - 8.0) | 0.20  (0.01 - 0.32) | 43.8  (36.1 - 46.5) | 0.9  (0.1 - 2.4) |
| Litletind,  Narvik | 3.8  (1.0 - 8.0) | 0.11  (0.04 - 0.28) | 37.6  (29.3 - 46.0) | 1.3  (0.4 - 3.9) | 3.5  (1.0 - 9.5) | 0.11  (0.06 - 0.17) | 45.6  (38.2 - 48.1) | 1.4  (0.5 - 4.1) |
| Storskartoppen, Narvik | 6.5  (0.3 - 18.1) | 0.16  (0.06 - 0.34) | 45.4  (32.9 - 48.9) | 3.5  (0.2 - 15.2) | 3.5  (1.7 - 8.5) | 0.16  (0.09 - 0.21) | 44.3  (40.0 - 50.2) | 2.2  (1.1 - 5.6) |
| Heia,  Lavangen | 4.0  (2.0 - 18.5) | 0.18  (0.11 - 0.37) | 43.7  (39.5 - 49.1) | 3.7  (1.5 - 15.7) | 14.8  (7.5 - 22.0) | 0.15  (0.13 - 0.21) | 48.2  (46.6 - 48.7) | 11.8  (5.9 - 14.4) |
| Gámariehppi, Lavangen | 1.3  (0.2 - 3.0) | 0.20  (0.09 - 0.43) | 34.7  (21.4 - 44.5) | 0.9  (0.1 - 1.8) | 0.4  (0.1 - 0.6) | 0.25  (0.07 - 0.36) | 25.8  (19.5 - 37.8) | 0.1  (0.1 - 0.3) |
| Rundkollen, Salangen | 1.0  (0.1 - 2.5) | 0.22  (0.09 - 0.41) | 37.9  (19.6 - 44.8) | 0.7  (0.1 - 1.5) | 1.0  (0.5 - 15.0) | 0.15  (0.01 - 0.38) | 38.3  (23.4 - 46.1) | 0.6  (0.5 - 1.4) |
| Kvitfjellet,  Senja | 6.5  (1.5 - 15.4) | 0.14  (0.06 - 0.32) | 46.2  (32.4 - 49.3) | 3.3  (1.3 - 9.4) | 1.0  (0.5 - 4.0) | 0.19  (0.06 - 0.29) | 41.9  (31.7 - 45.4) | 1.0  (0.3 - 1.7) |
| Suohpavuopmi, Senja | 10.0  (1.0 - 23.5) | 0.14  (0.08 - 0.34) | 46.02  (37.9 - 48.9) | 5.0  (1.4 - 13.5) | 5.8  (1.0 - 8.0) | 0.15  (0.11 - 0.23) | 47.2  (43.2 - 48.5) | 4.2  (1.0 - 5.1) |
